# Wounds and Wonder: Emotion, Imagination and War in the Cultures of Romantic Surgery

**DOI:** 10.1111/1754-0208.12684

**Published:** 2020-02-17

**Authors:** Michael Brown

**Affiliations:** ^1^ University of Roehampton, Humanities, Roehampton Lane, London, US and Canada only United Kingdom SW15 5PU

**Keywords:** Surgery, war, emotions, imagination, John Bell, Charles Bell, Waterloo, Romanticism

## Abstract

Abstract: This article uses the writings of the surgical siblings John and Charles Bell to explore the relationships between surgery, war and emotion in the Romantic era. Drawing on the argument that it was in this period that war came to be constructed as the ‘ultimate' emotional experience, rich in pathos and distinct from anything in civil life, it argues that surgeons such as the Bells might capitalise on the cultural cachet of war to bridge the professional and experiential divide between the civil and military spheres, but that this process was fraught with complexity and ambiguity, both politically and emotionally.

## I Introduction

When the Scottish surgeon Charles Bell (1774‐1842) heard the news of the victory at Waterloo on 22 June 1815, he exclaimed to his brother‐in‐law and fellow surgeon John Shaw (1792‐1827): ‘Johnnie! How can we let this pass? Here is such an occasion of seeing gun‐shot wounds come our very door, Let us go!'^1^ Charles was not the only British surgeon to seek practical experience and patriotic duty in the makeshift hospitals of Brussels and Antwerp; nor was he alone in recording his experiences. Even so, his are perhaps the most well known. In part, this is because his letters were later published, whereas those of most other correspondents are accessible only in manuscript form.^2^ But they also owe a not inconsiderable portion of their fame to their depth of sentiment. Charles's brother George Bell (1770‐1843) passed one of his letters on to Sir Walter Scott (1771‐1832), who was so moved by its emotional evocations (reading it ‘set me on fire', he claimed) that it inspired his own journey to the Continent, as well as his later semi‐fictional account of the aftermath of Waterloo, *Paul's Letters to his Kinsfolk* (1816).^3^ Moreover, Charles's time in Belgium also gave rise to another remarkable emotional ‘archive': namely the sketches and subsequent watercolours that he produced of some of the wounded soldiers he treated there.^4^

Charles's Waterloo paintings have received extensive attention from historians of art, including recent work by Aris Sarafianos and Anthea Callen, who use them to explore visual representations of pain and the sublime and of the male body, respectively.^5^ This art‐historical interest in Charles derives not only from the power of his images but also from the fact that he was deeply invested in art theory and practice and, in 1807, sought election to the post of professor of anatomy at the Royal Academy of Arts, albeit unsuccessfully.^6^ His war experiences, by contrast, have been rather less well studied by historians of medicine and surgery, most of whom have been concerned with his broader contributions to surgical education and to the disciplines of neurology and physiology.^7^

Ironically, perhaps, the fact that Charles Bell is deemed to have made a greater cultural impact than many of his surgical contemporaries has tended to obscure, rather than highlight, his professional identity, as many scholars have come to him with other purposes in mind. For example, by far the most significant account of his experiences in Brussels can be found in the work of Philip Shaw, who seeks to recover Charles's conception of suffering in relation to Waterloo's almost instant mythologisation as an act of ‘sublime labour' in the service of the nation.^8^ Shaw's reading of Charles's images and words is both sensitive and astute, capturing nuance and ambiguity. Despite its sophistication, however, Shaw's arguments might yet be developed, for his analysis, like much art‐historical scholarship on the subject, leaves Charles's professional identity somewhat opaque. For example, while he traces the complexity of Charles's responses to the suffering he witnesses, Shaw tends to characterise his surgical disposition as one of ‘detachment', setting Charles's emotionality and his identity as a surgeon in something of a tension.^9^

This article builds on Shaw's insights by employing an emotions history framework. It does this in two key ways. First, it situates Charles's writings and experiences, as well as those of his older brother and mentor, John Bell (1763‐1820), within the broader cultures of what I call ‘Romantic' surgery.^10^ The ‘Romantic' is a complex term that has been widely applied, including in Shaw's work. In terms of surgery, it describes an early nineteenth‐century professional culture that drew heavily on forms of sensibility that increasingly emphasised emotional intensity and authenticity. Together with the power of imagination, this allowed for a deeply intersubjective engagement with the patient's sufferings which shaped the therapeutic encounter in profound ways.^11^ What is also characteristic about this Romantic culture is the importance of emotional *experience* and the extent of emotional introspection involved in the shaping of surgical identities.^12^ At the turn of the century, for example, John Bell challenged the established stereotype of the rough 'sawbones', developing contemporary critiques of sensibility and artifice to shape an image of the surgeon as a dedicated professional whose emotional authenticity was rooted in the embodied experience of operative practice.^13^

In this way it is possible to move beyond the notion that detachment was the defining characteristic of contemporary surgery and to recognise the centrality of emotional experience and emotional expressiveness to Romantic surgical culture. But what this article is more especially concerned to do is to argue for the place of war, and its associated forms of sentiment, within that culture. John's wartime experiences are less well known than those of his brother, but he was also deeply invested in the treatment of wounded servicemen. Crucially, neither man was a military surgeon per se. While Charles wrote in 1807 that ‘of all things I should like to be kept and sent to the armies as a surgeon' and while John agitated for a role in the training of military surgeons, neither had served in the army or navy, and neither had any direct experience of battle.^14^ And yet, in their work, both men *imagined* themselves as battlefield surgeons, reflecting and capitalising on the experience of war. The use of the term ‘capitalise' is deliberate, for this article suggests that both men harnessed the emotional and cultural capital of war to shape their identities as surgeons.

There has been a remarkable growth of interest in the emotional history of war in recent years, and undoubtedly one of the most important contributions has been that of Yuval Noah Harari, who argues that, in the Romantic period, war came to be constructed as the ‘ultimate' emotional experience, rich in pathos and distinct from anything in civil life.^15^ Harari has his critics, not least among early modern historians who have argued for the emotional richness and complexity of war experiences in earlier centuries.^16^ Indeed, Bettina Noak suggests that early modern battlefield surgeons such as Amboise Paré (1510‐1590) also expressed sympathy for the sufferings of their patients.^17^ Nonetheless, even these historians concede that earlier accounts ‘may disappoint the historian who hopes to find close descriptions of the inner experiences and thoughts of the combatant' and recognise that the Seven Years War (1756‐63) and, more especially, the Revolutionary and Napoleonic Wars (1792‐1815) constitute a watershed in the emotional conceptualisation of war experience.^18^ The latter has been called the first ‘total war' and, particularly among the continental belligerents, involved a vast number of citizen soldiers on a scale hitherto unimaginable.^19^ At the same time, however, Harari has argued that this period saw the widening of the conceptual and experiential gulf between the civilian and military worlds, as the ‘flesh‐witnessing' of war was cast in increasingly ineffable terms.^21^ If Harari's observations are more problematic for the Continent's mass armies, they are less so for Britain, which fought the wars with a relatively small professional army.^21^ Even so, while this article acknowledges the importance of flesh‐witnessing, it also accords with the more nuanced readings of Shaw and Catriona Kennedy, which suggest that this gulf could be bridged through a shared culture of sentiment and sensibility, and through acts of imaginative identification.^22^ For civilian surgeons such as the Bells, therefore, the treatment of wounded soldiers and sailors held powerful emotional and patriotic appeal and was used to assert and sustain forms of cultural and professional authority.

This engagement with the wounded was not without its ambiguities, nor was it without personal emotional consequence. Charles Bell occupies a prominent place in the history of the emotions, not least because of his *Essays on the Anatomy of Expression in Painting* (1806). Indeed, Thomas Dixon has credited him with being the ‘coinventor' of the modern concept of emotions.^23^ Ironically, however, his own emotional character has been less well explored. Hence, the second major aim of this article is to analyse Bell's experiences of Waterloo in order to demonstrate the complex relations between emotional expressions and lived experience, as well as the ways in which these shape and sustain personal and professional identities. Here we must acknowledge the thorny issue of phenomenology. It would be naive to suggest that surgeons' lived emotions can be read off the page, and that their pronouncements on these matters can be taken at face value. And yet it would be equally reductive to suggest that they sought merely to exploit the sentimental value of the war wounded for their own professional ends. In order to reach a more nuanced understanding, then, this article adopts the model of the ‘emotive' outlined by William Reddy.^24^ In Reddy's conception, emotives are speech acts which not only have a ‘relational' quality (i.e., they communicate meaning) but also shape experience (they are ‘self‐altering'). For Reddy, the permissibility of emotional expression is determined by the dominant ‘emotional regime' of the period, which in this case was Romantic sentimentalism. But, as Reddy suggests, discrepancies in intentionality (or ‘goal conflict') can produce ‘emotional suffering'. Reddy's conception of ‘goal conflict' and ‘emotional suffering' can be quite narrow: for example, he often uses the example of political torture.^25^ Elsewhere, however, he posits a more expansive model in which emotional suffering might derive from a discrepancy between the inward feelings one experiences and one's ability to express those feelings in accordance with the conventions of the emotional regime; it is this form of emotional suffering that is particularly present in Charles's writings.^26^ It is important to note, however, that Charles's suffering did not derive from a goal conflict between surgery and sentiment per se. He often articulated the distress occasioned by the emotional demands of surgery, but such expressions were, as we have suggested, in total accord with the emotional conventions of Romantic surgery. What suffering he endured was, rather, the product of *war*, reconciling its horrors, and scale of suffering, with the conventions of Romantic sentimentalism and popular nationalism.

Reddy's conception of the emotive can thus significantly enhance our understanding of Charles Bell's complex response to the experience of treating the wounded. When confronted by the flesh‐witnessing of war, his language fails him and he is consumed by uncertainty and doubt. His was not a simple act of emotional withdrawal, or even simply a posture of ‘wearied detachment'.^27^ Rather, he was a man whose predominant concern was his inability to express himself fully, to navigate between his inward feelings and the conventions of sensibility. The word ‘wonder' in the title of this article thus refers not simply to the oft‐acknowledged sublimity of war but also to the doubts and anxieties produced by the reconciliation of experience with meaning. This might appear to be a failure on Charles's part, and was certainly productive of emotional suffering. However, this article suggests that it was also a kind of performative reflexivity, that the possession of feelings whose depth and intensity defied expression could actually serve as a marker of the most refined sensibility and, hence, of professional virtue.

## II John Bell: Mobilising the Wounded

For the most part, John Bell has never inspired quite the same degree of scholarly interest as his younger brother.^28^ He was born in 1763, the son of an Episcopalian clergyman, William Bell (1704‐1779), and was one of four brothers, two of whom – Robert (*c*.1760‐1816) and George – became advocates. Having trained as a surgeon at some expense, John was, in turn, required to educate Charles. As we shall see, they had a difficult relationship. George described John as a man of ‘restless vanity', writing that there ‘was in that man something uncomfortable and dispiriting to his friends'.^29^ Despite this, John was almost universally lauded as a surgeon and anatomist and became widely reputed as ‘the reformer of Surgery in Edinburgh, or rather the father of it'.^30^

John's opening of an anatomical school in 1790 was prescient, for three years later Britain would be at war with Revolutionary France and would require an increasing number of military surgeons. John had no training in military surgery, although he showed an early interest in the treatment of wounds that would have set him in good stead with his pupils, many of whom would go on to work for the Army Medical Department or the Navy's Sick and Hurt Board. In 1795 he published *Discourses on the Nature and Cure of Wounds*, which, like the rest of his work, is shaped by sentiment and exhibits a peculiar degree of emotionality and reflection, even by the highly expressive standards of the day.^31^ John's conception of the emotional intersubjectivity between patient and practitioner is a particularly powerful presence in this work. ‘Discourse IV', for example, concerns ‘Gun‐Shot Wounds', and the first part is dedicated to the examination of the wounded solider:

No sooner does the surgeon see his wounded soldiers carried into his tent, than the very sight of a man, pale and perhaps bleeding, awakens the strongest interest, and a lively anxiety, to know the nature of his wound; but how much stronger must the patient's own feelings be, who waits in awful suspence [*sic*], while he learns even from the countenance of his surgeon, the sentence of life or death!^32^

Here John imagines the surgeon's ‘lively anxiety' on seeing the wounded soldier, then imagines the soldier's own feelings of anxiety. In turn, these feelings are conceived not simply as the soldier's affective response to his wounded state but as a perceptual consequence of the surgeon's mood, as read from his face. This emotional reflection is critical to understanding John's conception of surgical practice. In this period surgeons often found it difficult to account for a patient's failure to survive an operation, or to recover from it, in terms other than the emotional. A patient who was reassured by his surgeon might come through a difficult procedure, whereas one who was alarmed and apprehensive might sink and die under even the most trivial complaint.^33^ In his advice concerning the surgical handling of wounded soldiers, John is thus at pains to recommend a compassionate and sensitive demeanour.

In addition to its depth of sentiment, what is remarkable about this passage is that John had never been a military surgeon. His projection of himself onto the battlefield, anxiously awaiting the arrival of *his* men into *his* tent, is therefore an act of pure imagination. At one level this act of imagination served a didactic purpose. After all, John was providing young men with exactly the kind of practical surgical education necessary for military service, and his book was clearly aimed at those seeking knowledge of injuries most commonly encountered in war. But it is also vital to acknowledge the role of what Graham Dawson calls ‘phantasy' here.^34^ The military had long exerted an influence on men's sense of themselves, and Samuel Johnson famously remarked that ‘Every man thinks meanly of himself for not having been a soldier, or not having been at sea'.^35^ In times of war, such as had broken out two years prior to the publication of John's book, these tensions were especially pronounced, as civilian men consumed tales and images of heroic and manly acts in the service of the nation while playing little or no role themselves. The French wars, as Linda Colley and David Bell have shown, constituted a collective national experience that encouraged various forms of ‘popular commitment', most notably, in Britain, the volunteer movement.^36^ And, as Kennedy has argued, acts of imagination allowed civilians to identify with a war that they were ‘living through, but not in'.^37^ Furthermore, as Shaw has suggested, the image of the soldier suffering in the national interest rose to prominence in this period and assumed a particular cultural cachet.^38^

For a civilian surgeon like John Bell, therefore, the imagining of oneself into the position of a battlefield practitioner could sustain a culturally resonant masculine identity, one that sublimated the individual into a national endeavour, while also capitalising on the rich pathos of the suffering soldier. Such rhetorical strategies could also play a more explicitly political function. As Catherine Kelly's work on the Army Medical Department has shown, there was no strict demarcation between the worlds of civilian and military medicine in this period. Regimental and ships' surgeons were, until later in the nineteenth century, effectively civilians in uniform and held generally low status. And yet, because the army and navy were among the few national institutions that could provide medical practitioners with a salaried living, the patronage and largesse of government officials were avidly sought.^39^ For many surgeons, the ideal was to secure a high‐ranking position within the medical‐military hierarchy, thereby accruing its pecuniary and cultural rewards without actually having to undergo the routine drudgery and dangers of quotidian military service. It should perhaps come as little surprise, therefore, that, some three years after his publication of *Discourses on the Nature and Cure of Wounds*, John attempted to mobilise his expertise in pursuit of professional advancement.

In 1797 John gained valuable practical experience of war wounds through his attendance on the sailors and marines of Admiral Duncan's fleet in the aftermath of the battle of Camperdown. The following year he penned a letter to George Spencer, First Lord of the Admiralty, which was subsequently published as *Memorial Concerning the Present State of Military and Naval Surgery* (1800). This letter bemoans the poor quality and organisation of military surgical training, and makes a plea for the establishment of a ‘National School of Naval and Military Surgery'. John was at pains to point out that his motives were sincere, and, in a language that spoke to contemporary anxieties, both about the dangers of artifice and about threats to the established social and political order, he writes:

I [shall not] affect a sensibility I do not feel; sincerity and truth are the only apologies for this intrusion. I am not one of those unindustrious, idle, turbulent men, who delight in complaining, changing and reforming; but I will mention without reserve some things which are avowedly wrong in a department of the public service, inferior to none in importance […] no one who has ever seen even a little service, who is not inured to sights of misery, whose heart is not shut against the compunctious visitings of nature; no thinking nor feeling man will deny, that this department needs to be reformed.^20^

Here, as elsewhere, John is keen to figure himself a man of deep and unaffected sensibility whose interests are congruent with those of the nation and the public. But, needless to say, his letter is not entirely disinterested, as he clearly imagines himself appointed professor to the school whose establishment he recommends:

I have studied my profession with honest diligence, and have applied myself also to the study of Naval and Military Surgery with particular care. At one time, my Lord, I attended the wounded seamen in the hospitals of Sheerness and Yarmouth, with the humanity and industry of one who loves his profession, who, while he is employed in instructing others, is not unwilling to improve his own knowledge.^41^

Clearly, John's appeal for preferment rested as much on his emotional investment in the suffering of soldiers and sailors as it did on his expertise. Indeed, his letter is underwritten by a profound sense of pity at the fate of those exposed to the horrors of war without sufficient resources and training. In perhaps the most remarkable passage John once again effects an imaginative transportation into the position of a military surgeon. Suggesting that many ships left port with no surgical assistant on board, he conjures a vivid image of the calamitous consequences of such lack of provision:

Had those shots which passed so often through the cockpit and which have killed so many, who being already wounded, had retired to the place of safety! had one of those shots struck the surgeon; what must have been the condition of those who survived him! Inevitable death from wounds which are not deadly, is an awful sentence! who can bear it? Let the man of the most determined spirit, my Lord, think but of this! and, if he have not that disregard of life which deprives mere animal courage of all praise, let him say with what heart he can go into the midst of battle, where in a few moments all is horror, confusion, and dismay; where the danger of the hour makes no respect of persons; where the high and lowly are laid side by side, dead and dying! and the surgeon stands for a moment in his place, alone, fixed and motionless, with folded hands, in horror and deep astonishment at the situation in which he finds himself! ‘Can such things be, and you that do behold them still preserve the natural ruby of cheeks?'^42^

For all its rhetorical force, what John's letter demonstrates, alongside the centrality of emotion in Romantic surgical self‐presentation, is the limited power of imagination alone to sustain textual and personal authority when not underwritten by the flesh‐witnessing of experience. Soon after its publication John's letter was reviewed in the Tory loyalist periodical the *Anti‐Jacobin Review and Magazine*. The anonymous author mocks John for his ‘great humanity, his unaffected sensibility, his sincerity', and his ‘astonishing disinterestedness'. But what was especially irksome for this reviewer was that John had the temerity to pronounce on such matters based ‘on the mere power of imagination'. What knowledge of naval and military surgery John possessed was, he claims, derived from ‘a transient peep into the hospitals of Sheerness and Yarmouth', and he notes with incredulity that ‘our author once published a Treatise upon Gunshot Wounds before he had seen either Sheerness or Yarmouth, or had any knowledge whatsoever of the subject, except what he learned from reading, hearsay and his own imagination'. Ridiculing John's claims about the superiority of education to experience, the reviewer writes that ‘it must be owing to this most peculiar and singular discovery of acquiring experience, by previous knowledge, that our author, in order to write upon gunshot wounds, did not think it necessary to see practice in the army or navy'. ‘Had he wished to figure in the army', he concludes, he should have ‘offered his own services, and those of his pupils, to his Royal Highness the Duke of York'. Instead, he declares himself ‘neither fit nor willing to leave the soil where chance has rooted him' and seeks office through patronage.^43^

This withering response to John's letter clearly demonstrates the difficulties faced by civilian surgeons when seeking to mobilise the wounded for rhetorical effect. Certainly, no response was forthcoming from the Admiralty to his proposition. However, it would be too easy to take the judgement of the *Anti‐Jacobin Review* at face value and to assume that John's lobbying failed because it was cynically and transparently self‐interested. Patronage was the means by which careers in this period, surgical and otherwise, were made; supplication was an ever‐present fact of public life. There is equally no reason to assume that John's lack of direct military experience was in itself the reason for his lack of success. After all, Robert Jackson, who was a respected military surgeon, found that his proposal for ‘an army medical practical school' fell on similarly deaf ears.^44^ Moreover, when a concession was eventually made to the need for some kind of formal provision for military surgery, and the regius chair of military surgery was established at the University of Edinburgh in 1806, John Bell was passed over in favour not of a seasoned military veteran but of John Thomson, a man with even less military experience than him, and a significantly inferior reputation as an operative surgeon.^45^ John Bell's lack of preferment was as much the product of his combative personality and political isolation than of any particular rhetorical miscalculation. He had alienated many in the Edinburgh medical community through his highly public row with the university's professor of the practice of physic, James Gregory (1753‐1821). Thomson, on the other hand, was an astute networker with better political connections, especially in Whig circles.^46^ Even so, as we shall now see with the career of John's brother Charles, a surgical sensibility that combined imagination with direct experience of the pathos of war was apt to convey a greater degree of emotional and professional authority, albeit one that was not without its own ambiguities.

## III Charles Bell: Straddling the Civil–Military Divide

Charles's relationship with his older brother was not an easy one. By all accounts, John's behaviour towards him was more in keeping with his ‘pugnacious' and quarrelsome manner than with his literary persona as ‘a man of real feeling'.^47^ Charles wrote to his beloved brother George, on the occasion of John's death in 1820, that ‘He did dunch and press one' (meaning he could be difficult) and that ‘since I lived with him I have scarcely enjoyed what may be called conversation'. Even so, while the two of them were emotionally distant, they were intellectually extremely close. Charles assisted John in his teaching, and they worked together on several publications, notably *The Anatomy of the Human Body* (1793‐1804). Charles wrote, concerning his abortive biography of John, that ‘he was more than I have made him, and I *feel* so much as he *felt* that I think I have represented many things truly which might have been forgotten'.^48^

Charles certainly shared his brother's conception of surgery as an intensely emotional experience. Though he once claimed that ‘My hands are better for operation than any I have seen at work', he regularly expressed profound anxiety in advance of a procedure, telling George in 1824 that ‘I must do an operation to‐morrow, which makes me to‐day quite miserable […] I have […] the conviction […] that I am providing for a relay and continual supply of suffering'.^49^ He likewise shared John's interest in the treatment of wounds. Having moved to London in 1804, the following year he resolved to attend the wounded from Trafalgar, though ultimately the distance to Plymouth was too great. In 1809, however, he made it to the Haslar hospital in Gosport, where many of those wounded at Corunna had been taken. His letter to George from Gosport paints a complex emotional picture. Its opening lines evoke the frisson of the civilian newly exposed to military culture: ‘“Who goes there?” “A friend!” “Countersign?” “Spain!” “Pass, friend; all is well!” Such is the frequent call under my window.' Charles was clearly proud of his service and, on his return to London, writes: ‘I find myself a foot taller. Shame upon the fellows who did not take my example to learn their profession.'^50^

The body of Charles's letter, however, suggests a more ambivalent response to his ‘strange time' in Hampshire:

I wish I had written to you during my first sensations – these were, I trust, such as every good man should feel; they are blunted by repetition and I hate myself for being what I am – so mere a creature like the rest, going about my common affairs. I have muttered bitter curses and lamentations, have been delighted with the heroism and prowess of my countrymen, and shed tears of pity in the course of a few minutes. I find myself, dear George, in a situation unexpected and strange, such as I hope you may never see. I have stooped over hundreds of wretches in the most striking variety of woe and misery, picking out the wounded.^51^

Charles expresses his feelings in terms that are in keeping with contemporary cultural norms: the religious (‘bitter curses and lamentations'), the patriotic (‘heroism and prowess of my countrymen') and the sentimental (‘shed tears of pity'). And yet he also suggests that his ‘sensations' have somehow fallen short of the momentous emotional demands of the occasion, and that he has been unable to maintain the heightened sensibility of the ‘good man' in the face of unceasing suffering and the routines of practice (his ‘common affairs'). As we have seen, Charles was apt to express strong emotions about his operative practice, but he never castigated himself for his feelings in his letters, except for this instance. Charles's emotional disposition is thus not a conscious act of ‘professional detachment'. It is clearly seen as a failure and a source of emotional suffering: cause, indeed, to ‘hate' himself. Those around him are not exemplars of moral or professional conduct; they are ‘mere creatures', and it is to their level of insensibility that he fears he has sunk. But, at the same time, Charles's very ability to judge his emotions (and to find them wanting) testified to his emotional acuity and high ideals, as much as to the dangers posed by the ‘blunting' effects of wartime suffering.

As we shall see, it would be a mistake to assume here a simple emotional disconnect between the civil and military surgeon, between the wide‐eyed innocent and the jaded veteran, not least because military surgeons also frequently expressed themselves in the language of sentiment and sensibility. And yet Charles's letter does hint at the ambiguities inherent in his straddling of the divide between the domestic and the ‘ unexpected and strange' circumstances of war. A similar pattern is evident in his subsequent work with the wounded of Corunna. In his *Dissertation on Gun‐Shot Wounds* (1814) he observes that

It is too common an opinion with surgeons in domestic practice, and in hospitals here at home, that there is nothing peculiar in gun‐shot wounds. It is mortifying to the pride of theory to see how often it is humbled before the conviction of practice; even the scenes I have witnessed, and the cases I have had under my care, have proved to me that the books we possess upon the subject of field‐practice do not even hint at the nature of the difficulties the surgeon has to encounter there. In the nature and in the progress of gun‐shot wounds, there is much to be observed which never is to be seen in domestic practice.^52^

Here Charles seeks authority in his own experience. However, like his brother, he also uses imagination to bridge the divide. At various points in the *Dissertation* he writes of things he has not seen, including surgical practice on a ship‐of‐the‐line:

When the drum beats to quarters, the surgeon has to see that the operation table is securely fixed; that there is sufficient supply of candles and lanterns, and that the assistants know their places […] It is now a time of fearful expectation, and there are few situations in which a man more requires coolness of reflection. It is now that he feels how much the nature of the wounds of those who may be brought down to him ought to have occupied his mind in previous study.^53^

Like his brother, Charles imagines not simply the practical aspects of military surgery but also its experiential dimensions and affective demands. The same is true of his observations on army service, where he writes of the surgeon's patients ‘thrown in crowds into churches and convents' or ‘[lying] in the streets of a town, as after a great engagement'.^54^

**Figure 1:**
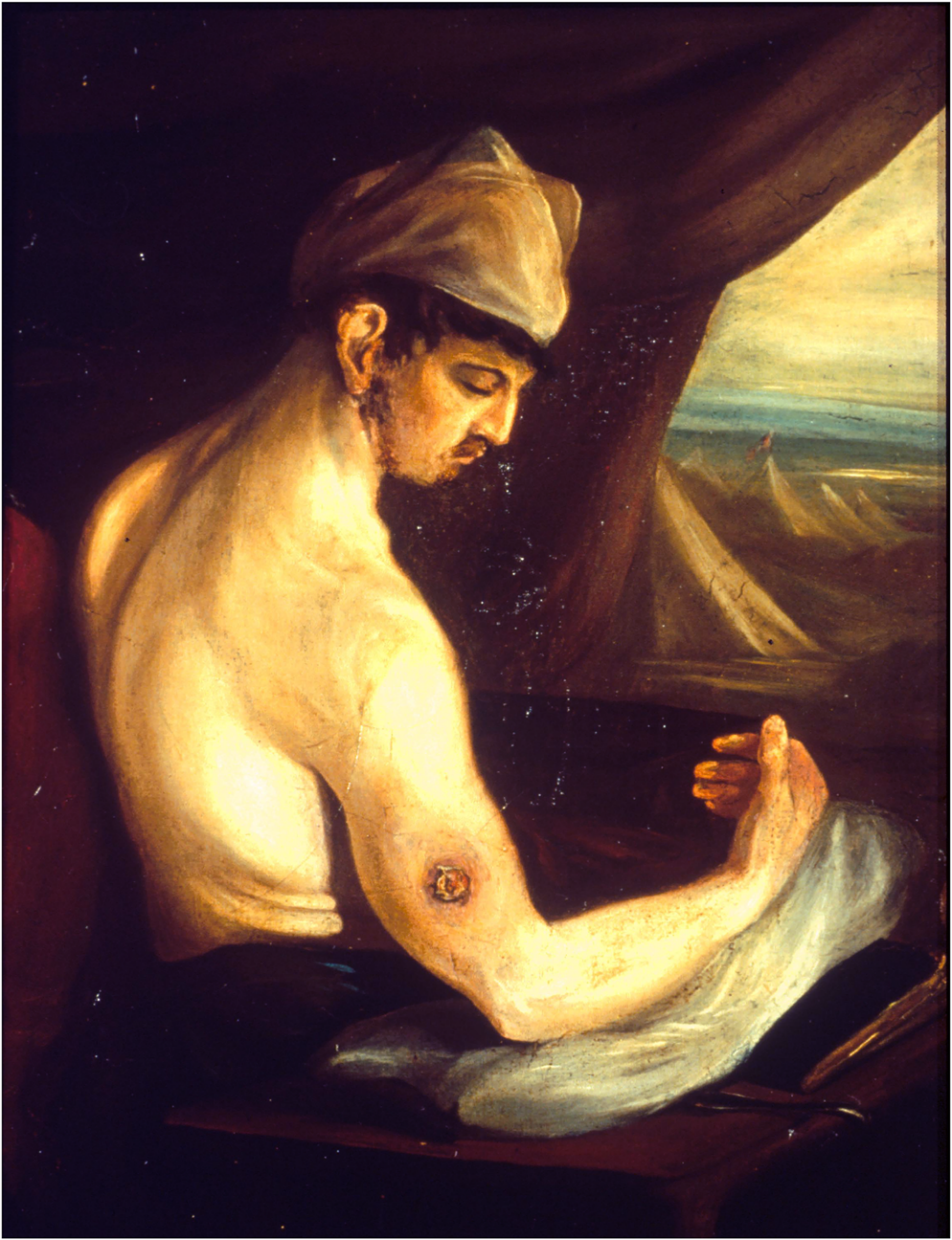
Charles Bell, *The Wounded following the Battle of Corunna: Musket Ball Wound of Humerus*, 1809, oil on canvas, 33.8 x 23.5 cm, Royal College of Surgeons of Edinburgh.

This imaginative projection onto the front line can be glimpsed in Charles's oil paintings of his ‘gun‐shot men'. Most of these simply represent the individual or their wound. Fig. 1 is unusual in that Charles has painted the figure in a tent, looking out onto rows of other tents in a military encampment. Without appropriate documentary evidence it is impossible to rule out the possibility that this is an accurate representation of arrangements at Haslar. However, given that Haslar was a permanent structure, it seems more likely that Charles's painting locates his patient, and thereby himself, in the imagined environs of Corunna, much as John imagined himself into the battlefield tent of the regimental surgeon.

Like his brother, Charles was clearly enamoured of military surgery's cultural cachet. He found his experiences with the war wounded to be intellectually rewarding and was flattered by the attention and respect he received from military surgeons, writing ‘I am doing myself much good by being so much among the army surgeons; whenever there is anything going on, they find me'.^55^ At the same time, he was also critical of military surgical practice. Charles's paintings are accompanied by annotations, such as ‘the method followed by our army surgeons was too bold' or that this was ‘the first [case] that suggested to me, the improper rule upon which our surgeons were proceeding'.^56^ Unlike his brother, however, Charles was somewhat more cautious in his attempts to capitalise on the experiences of war and to lecture military surgeons from a position of metropolitan safety. Indeed, in the *Dissertation* he offers something of an apology for his intrusion:

We enter on a subject which is very important and difficult, and even of some delicacy. Every young surgeon, when he finds himself in the field, expresses wonder that he so poorly conceived the nature of gun‐shot wounds from reading and the instruction of his teachers; and he adds, what can a lecture in a London theatre teach of this? When the same gentleman returns to us familiar with the horrors of the scene he has witnessed, and proud of the dangers and difficulties he has passed, he feels the subject his own, and an attempt like this, perhaps an encroachment.^57^

However, Charles argues that his was a worthy exercise precisely because military surgeons were unwilling to share their experiences:

But when with every sentiment of respect for practical knowledge thus honourably acquired, we here at home seeks to profit by the knowledge of the military surgeon, we find it withheld, and that there is nothing written by them upon the subject. Their apology is that a man must see and not read […] I do not recollect to have learned much of gun‐shot wounds before seeing them; but by much reading on the subject, I was prepared with many questions.^58^

Here Charles seeks to justify one ‘at home' speaking *to* and *for* those who had acquired their knowledge ‘honourably' in service, by suggesting that preparatory education is a crucial adjunct to practical experience. In this respect he was more successful than John, whose declamatory writings were perhaps inclined to alienate military surgeons rather than to instruct them. Even so, the straddling of military and civil domains remained an awkward professional posture, one requiring apology. Moreover, it also had emotional ramifications, for if treating the wounded of Corunna challenged Charles's emotional equipoise, it would pale in comparison with the experience of Waterloo.

## IV Waterloo: Mapping the Emotional Landscape

Before considering Charles's experiences in Belgium, it is important to consider, if only briefly, the broader emotional cultures of medical and surgical practice in the aftermath of Waterloo. In so doing, it is vital not to draw too sharp a distinction between the responses of civilian and military surgeons to the experience of battle or to assume that detachment was a default position of military service. John Thomson was regius professor of military surgery at the University of Edinburgh by the time he travelled to Brussels in June 1815, but he was a civil surgeon who lacked any significant experience of war. Even so, his letters are marked by a relative lack of emotional insight regarding his work in Brussels and Antwerp.^59^ By contrast, the various letters from military surgeons present at Waterloo, some of them contained in Thomson's own archive, are rich in emotional expression.

For example, Donald Finlayson, who was assistant surgeon to the 33rd Regiment of Foot, wrote to Deputy Inspector of Hospitals William Somerville (1777‐1860) stating that he did not ‘regret the advice you gave me to enter the army, notwithstanding what I have lately seen of the horrors of war'. During the Walcheren campaign (1809) ‘I saw little of the effects of war. Here I see both war and its effects, and the actions it exerts both on the physical and the moral constitution of man.'^60^ Few surgeons in Belgium had seen as much action as Finlayson, who was with the 33rd Foot at both Quatre Bras and Waterloo itself. As with many such correspondents, his celebration of victory was tempered by reflection on the personal and emotional costs of war:

I am happy that our Countrymen in future ages will have an example to imitate – aye to emulate, when they think of this battle. – I hope the advantages that may be expected to result from it, will, together with the conduct of their killed relations, console individuals for their private losses. – The Nation must lament her loss, but rejoice at the event.^61^

For the most part, however, it is lamentation rather than joy that characterises Finlayson's account. ‘What misery war causes!' he concludes in his first letter, while in his second he struggles to make meaning from the battle:

Horrible as man is – it may teach man instruction. If it do not show the amiable traits of human character – it makes him understand the depth of his depravity. I have seen enough of it. Would to Heaven I had not seen it in our own selves! I could tell a tale of horror to cause the hardest heart … but let a veil be drawn over the perversion of mankind.^62^

Finlayson's letters recount two particularly poignant vignettes. The first involves Lieutenant Arthur Gore who, at the battle of Quatre Bras, pursued Finlayson from Nivelles in search of his regiment, stating that ‘he would not have lost this for anything'. ‘Some time after', Finlayson writes, ‘I saw him lying on his back with the upper part of his head shot away. The rest of his countenance was most pleasant. I never saw it look more so, He seemed asleep.' The second ‘affecting circumstance' concerned Captain John Haigh, who

was killed with a cannonball as he was most coolly and gallantly encouraging his men and directing them how to act. His brother Lieut[enant] Haigh was close by and saw him fall and his bowels all gush out. He exclaimed – ‘oh kill me with him!' I endeavoured to console him, and said it might soon be our own fate. – He has since had his wish fulfilled, being shot through the neck on the 18th and dying soon after.^63^

John Davy (1790‐1868), the brother of the chemist Humphry Davy (1778‐1829) and an army hospital assistant, had not, unlike Finlayson, been present at the battle itself, something he ‘very much' regretted, but he nonetheless provides an account of the situation in Brussels, which he describes as an ‘astonishing dream'. He writes evocatively of the ‘sublime feeling' occasioned by hearing the guns at Quatre Bras and Ligny ‘like distant thunder', of the ‘horrors of the scene' as the wounded flood into Brussels and of the intense fear occasioned by the approach of the French. He also writes of how, when news of victory was received, and the sun broke through the clouds, ‘Excessive joy took the place of despair':

All was enlivened, and the faces of the inhabitants as much as the face of the sky but I should except the wounded, and I trust those who attended them. For my own part seeing so much misery around me, I became indifferent to life, and in the high excitement in which I was little regard for self remained. The horrors of the Hospital continued and increased.^64^

Davy's and Finlayson's letters combine personal reflection with an attempt to encompass the momentous scale of the occasion, although both acknowledge the limited comprehension of the individual. By contrast, Harpur Gamble (1790?‐1865), a naval surgeon, writing to his friend the St Thomas's Hospital surgical pupil John Flint South (1797‐1882), provides a more intimate account of surgical practice in the days following the battle, one that places special emphasis on humanity and compassion in the face of suffering. He writes of his indignation at the rough handling of patients at the Annonciade hospital in Brussels. ‘Entering our extensive ward in which from 60 to 70 were accommodated,' he begins, ‘I was attracted by the cries of a poor fellow receiving his daily visit and dressing.' This man, who had been wounded by a ball entering his right breast, striking a rib and exiting below the scapula, had ‘an inflamed wound with puffy edges giving out a serious discharge'. ‘[H]ere', Gamble notes, ‘my feelings were not a little roused hearing the poor fellow (while begging the assistant to be tender) receive a hearty derision accompanied by an order to be quiet.' In the case of another patient with a wounded thigh and broken arm, ‘Eggerton', the surgeon, warned the patient of the ‘pain they were going to put him to but his requests for [MS damaged] water were as little attended to as his feelings'. ‘There is on[e] way in which this model of practice can be justified', Gamble claims, with not a little irony: ‘a certain [porti]on of pain being allotted to each individual the orderly was feeling toward this poor fellow to hasten his release by giving it to him quickly':

Eggerton swore he'd be d—d if the orderly laid a hand on him he knew what he was the assistant would be d—d if he would dress him then, here etiquet [*sic*] gave way to humanity I offered my services to support the arm and they were accepted with pleasure by the poor fellow but I did not know what a tryal [*sic*] I was subjecting myself to it required every quiescent faculty of my body to keep down the indignation struggling to rise at every tug the poor fellow suffered.^65^

Such accounts provide evidence of the centrality of emotion in military‐surgical accounts of Waterloo. In this they are in keeping with the broader historiography of military culture, which has demonstrated that officers, and even those from the ranks, increasingly conceptualised war as an act of personal revelation and were ‘ever more concerned with describing the inner experience of battle and their emotional and psychological response to what they had witnessed'.^66^ One of the key points of debate in this literature has been the degree to which such experiences could be communicated to others and the extent to which the French wars saw the hardening of conceptual boundaries between the soldier and the civilian. Certainly, the sentiments of men like Finlayson, who sought to ‘draw a veil' over their reflections, suggest a certain ineffability to military experience. But what of those civilians who saw the effects of war at first hand? Famously, there were many civilians present in Brussels at the outbreak of hostilities with France, but surgeons such as Charles Bell were in a unique position to experience the horrors of war from a civilian standpoint. As we shall see, this could present a set of complex emotive and expressive challenges which nonetheless point to the importance of sensibility in understanding Romantic surgical culture and identity.

## V Charles Bell and Waterloo: Expressing the Ineffable

As we have heard, Charles Bell was a man who conceived of surgery as an intensely emotional experience. He was also a man whose general outlook on life was shaped by deep religious conviction and a Romantic sensibility. In May 1807 he wrote to George to tell him of a revelation he had experienced after performing an operation on one of his patients in the country. Having woken early, he ran outside and leapt the garden wall, engaging every animal he encountered in conversation. He then returned to bed to enjoy a ‘waking dream' in which ‘all was right in the system of the universe' and whence he gained the conviction that without ‘suffering and disease […] we should have been inanimate, cold, and heartless creatures' and that humans had been ‘granted a still higher enjoyment in the contemplation of mind […] strengthened by communication and sympathy'.^67^ For Charles, this contemplation and sympathy could be a source of consolation and joy. However, it could also be a source of deep sorrow, such as after the premature death of John Shaw, when he would wake weeping in the night, exclaiming ‘it is *imagination* that kills me'.^68^

Charles's susceptibility to feeling (he once wrote that ‘I get wearied – exhausted by the sufferings of others'^69^) is powerfully evident in his writings in the aftermath of Waterloo. Shortly after his arrival in Brussels, he made the following comment in his notebook:

A little while I must write before going to bed. But how? – in such a crowd of images – after such fatigue – fatigue which I did not know I could have undergone. I have been chiefly in four great hospitals; but I must not speak of them, only of the town, as a common traveller. It would be natural to say, ‘how stupid: how supine'. But no! the excitement and the exertions have been the greatest possible; but it is past, and I must not wonder if I do not see what, as a newcomer, I feel. I must not wonder if I do that there are smiles, and that people transact their business as in common days. I understand that people have … Oh? It is too much – my head!^70^

This passage encapsulates the challenges intrinsic to the emotive, to the articulation of inward feelings in a culturally acceptable form. As such, it bears close reading. Made shortly before bedtime after a physically and emotionally exhausting day, Charles's jottings find him incapable of ordering the ‘crowd of images' into a meaningful and satisfactory narrative. Indeed, he is disinclined to ‘speak' of his experiences with the wounded at all, suggesting that he should restrict himself to the literary conventions of the sightseer. He is uncomfortable with his feelings, imagining that they might appear ‘stupid' and ‘supine', yet he asserts that the physical exertions and nervous excitements of the day have been ‘the greatest possible'. He tells himself that the gulf between his inward sentiments and the observed behaviour of others should come as little surprise (‘I must not wonder if I do not see what […] I feel'), something that he ascribes to his status as a ‘newcomer' to the battlefield. At the same time, however, he assures himself that when he observes suffering and experiences distress, he must not forget that there is happiness in the world, that there are ‘smiles' and that ‘people transact their business as in common days'. As in his experience at Haslar, Charles is acutely aware of the emotional space between the ‘common days' of ordinary life and the extraordinary conditions of war. As with his professional straddling of the civil‐military divide, which produces ambiguity, Charles is perpetually dissatisfied with his emotional calibration. At Haslar he hated himself for failing to maintain an acute sensibility in the face of extraordinary suffering. In Brussels he is nearly overwhelmed by it, and his attempts to reconcile his own sensations with the demeanour of others collapse into incoherence and mental disorientation.

In the rest of Charles's notebook, and in his letters, he is more eloquent and confident in recounting his experiences, and certainly more comfortable in his liminal identity as both tourist and surgeon. After attending the Allied wounded, he takes a tour of the battlefield, which presents the opportunity for a more conventionally pathetic meditation on the scattered letters of the French and Prussian dead. After this, and having determined that the ‘best cases, that is, the most horrid wounds, left totally without assistance, were to be found in the hospital of the French wounded', he spends several exhausting days tending to these men, many of whom have been lying on the battlefield for several days.^71^

Despite their comparative lucidity, however, Charles's letters hint at inward agonies only partly disguised by generic forms and sentiments. In his letter to George, for example, he oscillates between an admiration for the ‘Strong, thick‐set, hardy veterans' of the Grande Armée and a ‘detestation' of the ‘fierceness […] cruelty, and bloodthirstiness' of ‘this race of banditti'; yet he ends with reference to a wounded French woman and the observation that ‘It is dreadful to visit these wounded French' and to hear their plaintive cries and declarations of suffering.^72^ Likewise, in his letter to his wife, Marion Bell (1786?‐1876), his exquisite sentiment is evident in his account of a ‘pretty girl who stands to sing to a low organ sweet German airs'. ‘Oh you cannot think how I enjoy a little visionary scene, a little romance', he writes, adding that ‘nothing raises the fit so much as these charming foreign airs'.^73^

However, in his letter to his friend the Whig MP Francis Horner (1778‐1817) Charles's emotional tribulations rise once more to the surface of his writing. This letter was penned on his return to London, where he finds himself ‘engaged in my usual occupations, and consequently disengaged of the horrors of the battle of Waterloo'. ‘I feel relief in this', he continues, ‘for certainly if I had written to you from Brussels, I should have appeared very extravagant.' Once again, then, Charles is conscious of his liminality, of his crossing an experiential and emotional divide between the domestic and the martial, the ordinary and the extraordinary. And he is likewise conscious of the ineffable, untranslatable or, at the very least, discomforting quality of those feelings engendered by the horrors of war. Charles is not simply a witness: he is an actor transformed by his experiences, undergoing ‘an absolute revolution […] in my economy, body and soul'. He proceeds to tell Horner of his work with the French wounded, though he asserts that ‘it is impossible to convey to you the picture of human misery continually before my eyes'. He writes of being swamped by the number of wounded men:

All the decencies of performing surgical operations were soon neglected, While I amputated one man's thigh, there lay at one time thirteen, all beseeching to be taken next; one full of entreaty, one calling upon me to remember my promise to take him, another execrating.

Charles is conscious of the ‘decencies' that made civil surgery comparatively tolerable, and of their swift neglect in Brussels. He is also conscious of the embodied extremes of wartime surgery, stating that it ‘was a strange thing to feel my clothes stiff with blood, and my arms powerless with the exertion of using the knife'. Moreover, he speaks, in particular, to the unique emotional demands of wartime surgery:

[It was] more extraordinary still to find my mind calm amidst such variety of suffering; but to give one of these objects access to your feelings was to allow yourself to be unmanned for the performance of a duty. It was less painful to look upon the whole than to contemplate one object.^74^

Shaw reads this as a conscious act of detachment, the putting on of ‘armour' to ‘protect the core self from the intrusion of feminized affects'.^75^ However, this risks reading emotions anachronistically, as a contaminant of the professional persona. Charles does not represent his disposition as an act of masculine defiance or a rejection of affect. Rather, as at Haslar, he is surprised by his sensations, or rather by the fact that he is not entirely overcome by them. This man of sensibility, so easily moved in ordinary days, finds the experience of war and his ability to keep a calm mind amid such scenes of suffering equally ‘extraordinary'. Despite his use of the term ‘unmanned', there is nothing ‘feminized' about emotion here. Charles does not suggest that to feel would be womanish. Rather, in what reads almost like a *post hoc* justification for perceived insensibility, he suggests that being overwhelmed by sorrow or pity would prevent him from performing his duty. And the danger of being overwhelmed by the sufferings of the individual is precisely due to the acuity of his sentiment, not its absence.

Indeed, the emotional ambiguity of Charles's letter and its relation to his liminal identity are confirmed by the final section, for here it pauses, to be concluded, in this manner, the following day:

I was interrupted, and now I perceive I was falling into the mistake of attempting to convey to you the feelings which took possession of me, amidst the miseries of Brussels. After being eight days among the wounded I visited the field of battle. The view of the field, the gallant stories, the charges, the individual instances of enterprise and valour recalled me to the sense the world has of victory and Waterloo. But this is transient. A gloomy, uncomfortable view of human nature is the inevitable consequence of looking upon the whole as I did – as I was forced to do.

It is a misfortune to have our sentiments so at variance with the universal impression. But there must ever be associated with the horrors of Waterloo, to my eyes, the most shocking sights of woe, to my ear accents of entreaty, outcry from the manly breast, interrupted forcible expressions of the dying, and *noisome smells*.^76^

Again Charles alludes to the ineffable qualities of his experiences and to the ‘mistake' of trying to communicate to a civilian reader, who has not experienced such things, ‘the feelings which took possession of me, amidst the miseries of Brussels'. There is a suggestion, in that phrase, that those feelings are past, that they are left behind in Belgium. But in actual fact the emotional consequences of Waterloo continue to shape Charles, to produce in him ‘a gloomy, uncomfortable view of human nature'. Peter Stanley suggests that Charles was ‘one of the few surgeons to acknowledge the trauma he suffered', but this too shades into anachronism.^77^ As William Reddy's work suggests, emotives are both a performance and an experience, and in Charles's letter to Horner we have neither detachment nor trauma.^78^ What we have instead is the manifestation of an acute sensibility, one so sensitive that it transcends expression and must be carefully navigated lest it stray too far into despondency and away from the prevailing mood of national celebration. What we also have is a man shaped by his identity as a surgeon, standing at the threshold of the martial and the domestic, the ordinary and the extraordinary. He is a man transformed, a man who, unlike those civilians (‘the world') who will consume consolatory tales of individual heroism, is ‘forced', by the flesh‐witnessing of experience, to contemplate ‘the whole'.

**Figure 2:**
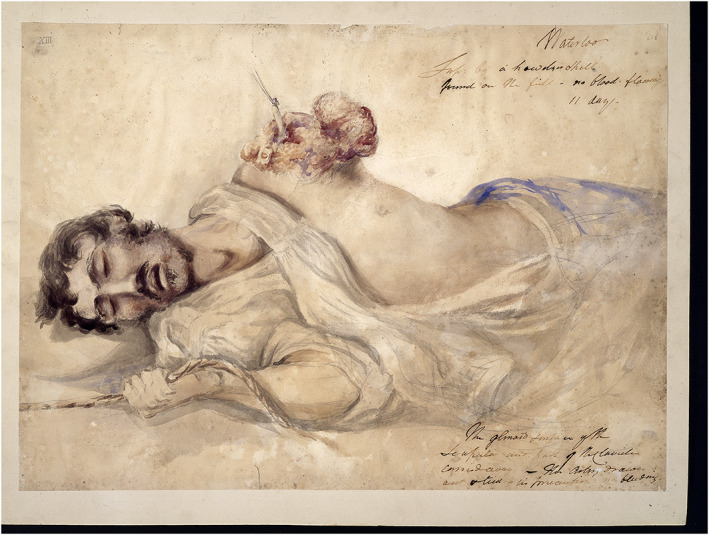
Charles's original sketches no longer survive, but this image shows one of the later watercolours based on them. It portrays a severe wound to the upper body of an anonymous British soldier, *c*.1815. Wellcome Library, London.

This ineffability of experience and its relation to an acute sensibility are central to understanding Charles's social and professional persona. He concludes his letter to Horner by stating that ‘I must show you my notebooks, for […] I took my notes of cases generally by sketching the object of our remarks' (Fig. 2). These images, he suggests, might serve as a form of virtual witnessing, communicating what he cannot adequately articulate in words. And they might also bridge the emotional gulf between the parlour and the battlefield and thereby ‘convey an excuse for this excess of *sentiment*'.^79^ Moreover, while it might be tempting to read ineffability as a function of trauma, a due consideration of Charles's character and correspondence suggests another, more fruitful, interpretation. In 1805 Charles wrote to George about a visit to the House of Common where he heard speeches by some of the leading figures of the day, including the prime minister, William Pitt the Younger (1759‐1806) and the playwright Richard Brinsley Sheridan (1751‐1816). His letter reflects on the relationship between manner and feeling, and asserts the superiority of ‘plain expression to ornament'. But his most revealing observation concerns the veteran radical Whig Charles James Fox (1749‐1806). ‘Having a headache from three long monotonous speeches', he wrote, ‘Fox's manner, by giving a new stimulus, cured me. His manner is that of a man who has *more within him than he can give utterance to, or find words to express*.'^80^

## VI Conclusion

Like Fox, Charles Bell had ‘more within him' than he could ‘find words to express'. In that moment in the House of Commons he recognised, in the absence of full expression, the essence of authentic sentiment. This is not to suggest that Charles sought to mimic Fox, or that his emotions were in any way mannered or performative, at least no more so than anyone else's. What it does suggest is that he saw something of value in Fox, something that spoke to his own sense of emotional depth and sincerity. From this we can gain an insight into the ways in which this particular Romantic surgeon conceived of his emotional identity, and of the relationship between language and lived experience. This was not an identity characterised by emotional detachment but, rather, one shaped by the values of sentiment and sensibility. And it was a relationship that bears out William Reddy's concept of the emotive as a dynamic interaction of inward sensations and social conventions.^81^

Of course, Charles's experiences were not those of most men. Few in this period were exposed to the horrors of battle, and fewer still shaped their professional identity, at least in part, through the care of those maimed and disfigured by war's cruelties. Indeed Charles was especially unusual in being a civil surgeon who had extensive experience of war wounds. In that regard, however, he provides a revealing case study, not simply of how war was experienced and articulated in the Romantic period but also of how those experiences and emotions were translated across the civil–military divide. Yuval Noah Harari has suggested that this period saw the widening of the gulf between civilians and soldiers.^82^ Others have suggested a more complex picture. What is evident from Charles's writings, and those of army surgeons who served at Waterloo, is not that civilians and soldiers were poles apart, either in terms of their emotional experiences or their recording of them (both of which were shaped by the cultures of sensibility), but that men like Charles were peculiarly conscious of their liminal identity and of the difficulties and ambiguities of moving between the civil and military spheres. For Charles, war was a ‘wonder', not only in terms of its sublime and extraordinary qualities but also because of the doubts and anxieties it could engender about one's emotional disposition, challenging his sense of what ‘every good man should feel'. What this suggests, is that, while not unbridgeable, the gulf between the soldier and the civilian was indeed widening in this period as the experience war was being constructed in increasingly transcendent terms.

At the same time, however, if the direct experience of battle now distinguished the military from the rest of society, this period also saw a significant increase in the wider social and cultural appeal of war. As Catriona Kennedy observes of he Revolutionary and Napoleonic Wars, they were a ‘conflict [that] was, in many ways, experienced through the imagination'.^83^ For the most part, this scholarly sensitivity to the imaginative appeal of war has focused on ‘scarlet fever', ‘naval [*sic*] gazing' or other aspects of the ‘pleasure culture of war'.^84^ What the example of John and Charles Bell demonstrates, however, is that it could also be used, with varying degrees of success, to buttress professional identity and authority. Through their work on war wounds, and their imaginative investment in the suffering solider, the Bells sought not only to carve out a professional niche but also, more broadly, to associate themselves with the values and virtues of military service. And in that sense they prefigured the medical and surgical investment in war and military heroism that would come to play a vital, though not unambiguous, role in the shaping of nineteenth‐century medical professional culture.^85^

## Notes

1
*Letters of Sir Charles Bell* (London: John Murray, 1870), p.227.

2 The original manuscript versions do not appear to survive. His letters to his brother were published, along with a selection of others, in 1870.

3 John Gibson Lockhart, *Memoirs of the Life of Sir Walter Scott, Bart*, vol. III (Edinburgh, 1837), p.347‐50. See also Graham Tulloch, ‘Walter Scott and Waterloo', *Romanticism* 24:3 (2018), p.266‐77.

4 For a facsimile of these paintings, see M. K. H. Crumplin and P. Starling*, A Surgical Artist at War: The Paintings and Sketches of Sir Charles Bell, 1809‐1815* (Edinburgh: Royal College of Surgeons of Edinburgh, 2005).

5 Aris Sarafianos, ‘Wounding Realities and “painful excitements”: Real Sympathy, the Imitation of Suffering and the Visual Arts after Burke's Sublime', in Tomas Macsotay, Cornelis van der Haven and Karel Vanhaesebrouck (eds), *The Hurt(ful) Body: Performing and Beholding Pain, 1600‐1800* (Manchester: Manchester University Press, 2017), p.170‐201; Anthea Callen, *Looking at Men: Art, Anatomy and the Modern Male Body* (New Haven, CT: Yale University Press, 2018), Chapter 5. Charles's work has also informed theatre studies, notably Matthew Wilson Smith, *The Nervous Stage: Nineteenth‐Century Neuroscience and the Birth of Modern Theater* (Oxford: Oxford University Press, 2017).

6 Carin Berkowitz, *Charles Bell and the Anatomy of Reform* (Chicago, IL: Chicago University Press, 2014), p.25‐30.

7 Berkowitz, *Charles Bell*, p.76. See also Michael J. Aminoff, *Sir Charles Bell: His Life, Art, Neurological Concepts, and Controversial Legacy* (Oxford: Oxford University Press, 2017), Chapter 5, which does discuss Charles's war experiences at more length but adds little to our understanding.

8 Philip Shaw, *Suffering and Sentiment in Romantic Military Art* (Aldershot: Ashgate, 2013). See also Philip Shaw, *Waterloo and the Romantic Imagination* (Basingstoke: Palgrave Macmillan, 2002), p.39‐43.

9 Shaw, *Suffering*, p.187, 189, 191.

10 Michael Brown, ‘Surgery and Emotion: The Era Before Anaesthesia', in Thomas Schlich (ed.), *The Palgrave Handbook of the History of Surgery* (London: Palgrave Macmillan, 2018), p.327‐47; Michael Brown, ‘Redeeming Mr Sawbone: Compassion and Care in the Cultures of Nineteenth‐Century Surgery', *Journal of Compassionate Healthcare* 4:13 (2017). For other references to Romanticism with regard to medicine and surgery, see James Robert Allard, ‘Medicine', in Joel Faflak and Julia M. Wright (eds), *A Handbook of Romanticism Studies* (Chichester: Wiley, 2012), p.375‐90.

11 For a general history of sensibility, see G. J. Barker‐Benfield*, The Culture of Sensibility: Sex and Society in Eighteenth‐Century Britain* (Chicago, IL: Chicago University Press, 1992). On Romantic sensibility, see Julie Ellison, ‘Sensibility', in Faflak and Wright (eds), *Handbook of Romanticism Studies*, p.37‐53. For the importance of emotional authenticity within Romantic sensibility, see Lionel Trilling, *Sincerity and Authenticity* (London: Oxford University Press, 1972), and Tim Miles and Kerry Sinanan (eds), *Romanticism, Sincerity and Authenticity* (Basingstoke: Palgrave, 2010).

12 For the move to an increasingly introspective, ‘inwardly‐turned', self in the later eighteenth century, see Dror Wahrman, *The Making of the Modern Self: Identity and Culture in Eighteenth‐Century England* (New Haven, CT: Yale University Press, 2004).

13 Michael Brown, ‘Surgery, Identity and Embodied Emotion: John Bell, James Gregory and the Edinburgh “medical war”', *History* 104:359 (2019), p.19‐41.

14 Charles Bell to George Bell, 21 May 1807, *Letters*, p.96.

15 Yuval Noah Harari, *The Ultimate Experience: Battlefield Revelations and the Making of Modern War Culture, 1450‐2000* (Basingstoke: Palgrave Macmillan, 2008).

16 For example, see Erika Kuijpers and Cornelis van der Haven (eds), *Battlefield Emotions, 1500‐1800: Practices, Experience, Imagination* (London: Palgrave, 2016).

17 Bettina Noak, ‘Emotions, Imagination and Surgery: Wounded Warriors in the Works of Amboise Paré and Johan van Beverwijck', in Kuijpers and van der Haven (eds), *Battlefield Emotions*, p.71‐91. However, aside from one example, Noak does not elaborate Paré's own emotional disposition and is predominantly concerned with the power of imagination as a tool of healing, rather than as a means of personal or professional self‐fashioning, as here.

18 Erika Kuijpers and Cornelis van der Haven, ‘Introduction', in Kuijpers and van der Haven (eds), *Battlefield Emotions*, p.11. Indeed, in the same introduction, the editors identify the mid‐eighteenth century as being ‘the time in which soldiers started to describe their own feelings in combat' (p.5) while several authors point to the *Diary of Ulrich Bräker* (1788‐9) in announcing these inwardly turned emotional narratives of war: for example, Marian Füssel, ‘Emotions in the Making: The Transformation of Battlefield Experiences in the Seven Years' War (1756‐1763)', p.149‐72.

19 David Bell, *The First Total War: Napoleon's Europe and the Birth of Modern Warfare as We Know It* (Boston, MA: Houghton Mifflin, 2007).

21 Harari, *Ultimate Experience*, Chapter 6.

21 Indeed, Christy L. Pichichero's *The Military Enlightenment: War and Culture in the French Empire from Louis XIV to Napoleon* (Ithaca, NY: Cornell University Press, 2017) argues for the elision of civilian and military worlds in eighteenth‐century France.

22 Shaw, *Suffering*; Catriona Kennedy, *Narratives of the Revolutionary and Napoleonic Wars: Military and Civilian Experience in Britain and Ireland* (Basingstoke: Palgrave Macmillan, 2013).

23 Thomas Dixon, ‘“Emotion”: The History of a Keyword in Crisis', *Emotion Review* 4:338 (2012), p.341.

24 For an equivalent use of Reddy's concept of the emotive, see Ian Germani, ‘Mediated Battlefields of the French Revolution and Emotives at Work', in Kuijpers and van der Haven (eds), *Battlefield Emotions*, p.173‐94.

25 William Reddy, *The Navigation of Feeling: A Framework for the History of the Emotions* (Cambridge: Cambridge University Press, 2001), Chapters 3 and 4, particularly p.128‐9.

26 Jan Plamper, ‘The History of Emotions: An Interview with William Reddy, Barbara Rosenwein, and Peter Stearns', *History and Theory* 49 (May 2010), p.237‐44.

27 Shaw, *Suffering*, p.191.

28 For the exception, see Brown, ‘Surgery, Identity'. See also: E. W. Wallis, ‘John Bell, 1763‐1820', *Medical History* 8:1 (1964), p.63‐9; K. Grudzien Baston, ‘Bell, John (1763‐1820)', in H. C. G. Matthew and B. Harrison (eds), *Oxford Dictionary of National Biography* (Oxford; Oxford University Press, 2004); and M. H. Kaufman, ‘John Bell (1763‐1820), the “Father” of Surgical Anatomy', *Journal of Medical Biography* 13:2 (2005), p.73‐81.

29 Royal College of Surgeons of Edinburgh, GD82, Bell family archive, Box 1/2, handwritten notes and memoranda by George Joseph Bell (1770‐1843), fol. 1.

30 John Struthers, *Historical Sketch of the Edinburgh Anatomical School* (Edinburgh, 1867), p.43.

31 For the ‘standards of the day' in surgery see Brown, ‘Surgery and Emotion', Brown, ‘Redeeming Mr Sawbone', and Brown ‘Surgery, Identity'.

32 John Bell, *Discourses on the Nature and Cure of Wounds* (Edinburgh, 1795), p.177.

33 Brown, ‘Surgery and Emotion', p.337.

34 Graham Dawson, *Soldier Heroes: British Adventure, Empire and the Imagining of Masculinities* (London: Routledge, 1994). Dawson derives the term from the psychoanalytic work of Melanie Klein.

35 R. W. Johnson (ed.), *Selections from James Boswell's Life of Samuel Johnson* (Oxford, 1823), p.147. On the interrelationships between civilian and military masculinities in this period see Joanne Begiato, *Manliness in Britain, 1760‐1900: Bodies, Emotion and Material Culture* (Manchester: Manchester University Press, 2020). See also Karen Downing, *Restless Men: Masculinity and Robison Crusoe, 1788‐1840* (Basingstoke: Palgrave Macmillan, 2014).

36 Linda Colley, *Britons: Forging the Nation, 1707‐1837* (New Haven, CT: Yale University Press, 1992); Bell, *First Total War*.

37 Kennedy, *Narratives*, p.25, 161‐78.

38 Shaw, *Suffering*.

39 Catherine Kelly, *War and the Militarization of British Army Medicine, 1793‐1830* (London: Pickering and Chatto, 2011).

20 John Bell, *Memorial Concerning the Present State of Military and Naval Surgery* (London: 1800), p.2.

41 Bell, *Memorial*, p.3.

42 Bell, *Memorial*, p.11‐12.

43
*Anti‐Jacobin Review and Magazine* 29:7 (1800), p.293‐300.

44 Matthew H. Kaufman, *The Regius Chair of Military Surgery in the University of Edinburgh, 1806‐55* (Amsterdam: Rodopi, 2003), p.53‐4.

45 Kaufman, *The Regius Chair*, p.47‐66.

46 On the Gregory row, see Brown, ‘Surgery, Identity'. For a study of Thomson, see L. S. Jaycna, *Philosophic Whigs: Medicine, Science and Citizenship in Edinburgh, 1789‐1848* (London: Routledge, 1994).

47 Henry Cockurn, *Journal of Henry Cockburn: Being a Continuation of the Memorials of his Time, 1831‐1854*, vol. II (Edinburgh: 1874), p.203; Bell, *Nature*, p.27.

48 Charles Bell to George Bell, 12 August 1820, *Letters*, p.267‐8 (emphasis added).

49 Charles Bell to George Bell, 8 December 1835, *Letters*, p.346; Charles Bell to George Bell, 28 July 1824, *Letters*, p.285.

50 Charles Bell to George Bell, 3 February 1809, *Letters*, p.139; Charles Bell to George Bell, ‘Wednesday', *Letters*, p.140.

51 Charles Bell to George Bell, 3 February 1809, *Letters*, p.139.

52 Charles Bell, *A Dissertation on Gun‐Shot Wounds* (London: Longman, Hurst, Rees, Orme and Brown, 1814), p.3‐4.

53 Bell, *A Dissertation*, p.5.

54 Bell, *A Dissertation*, p.7.

55 Charles Bell to George Bell, 30 May 1809, *Letters*, p.152.

56 Crumplin and Starling, *Surgical Artist*, p.7‐8.

57 Bell, *A Dissertation*, p.33‐4.

58 Bell, *A Dissertation*, p.34.

59 National Library of Scotland , MS 9326, Thomson family correspondence.

60 Thomson family correspondence, fol. 45.

61 Thomson family correspondence, fol. 46.

62 Thomson family correspondence, fol.47v.

63 Thomson family correspondence, fol. 48v.

64 Thomson family correspondence, fols 68‐69.

65 Royal College of Surgeons, London, MS0232/8, letters to John Flint South from family and friends (unpaginated).

66 Kennedy, *Narratives*, p.72. See also, Harari, *Ultimate Experience*, Chapter 6.

67 Charles Bell to George Bell, 11 May 1807, *Letters,* p.94‐5.

68 'Lady Bell's Recollections', *Letters,* p.406.

69 Charles Bell to Marion Bell, 8 August 1841, *Letters*, p.392.

70 ‘Brussels diary’, *Letters*, p.229‐30.

71 ‘Brussels diary’, *Letters*, p.232‐8; Charles Bell to Francis Horner, July 1815, *Letters*, p.246.

72 Charles to George Bell, 1 July 1815, *Letters*, p.240‐43.

73 Charles Bell to Marion Bell, 2 July 1815, *Letters*, p.244.

74 Charles Bell to Francis Horner, July 1815, *Letters*, p.246‐7.

75 Shaw, *Suffering*, p.194‐5.

76 Charles Bell to Francis Horner, July 1815, *Letters*, p.248 (emphasis in the original).

77 Peter Stanley, *For Fear of Pain: British Surgery, 1790‐1850* (Amsterdam: Rodopi, 2003), p.120.

78 On the problematic uses of ‘trauma' see Tracey Loughran, ‘Shell Shock, Trauma, and the First World War: The Making of a Diagnosis and its Histories', *Journal of the History of Medicine and Allied Sciences* 67:1 (2012), p.94‐119.

79 Charles Bell to Francis Horner, July 1815, *Letters*, p.248.

80 Charles Bell to George Bell, 24 June 1805, *Letters*, p.49 (emphasis added).

81 Reddy, *Navigation of Feeling*.

82 Harari, *Ultimate Experience*.

83 Kennedy, *Narratives*, p.161.

84 Louise Carter, ‘Scarlet Fever: Female Enthusiasm for Men in Uniform, 1780–1815', in Kevin Linch and Matthew McCormack (eds), *Britain's Soldiers: Rethinking War and Society, 1715‐1815* (Liverpool: Liverpool University Press, 2014), p.155‐80; Kennedy, *Narratives*, Chapter 7.

85 Michael Brown, ‘“Like a Devoted Army”: Medicine, Heroic Masculinity and the Military Paradigm in Victorian Britain', *Journal of British Studies* 49:3 (2010), p.592‐622.

